# A survey and stakeholder consultation of Independent Domestic Violence Advisor (IDVA) programmes in English maternity services

**DOI:** 10.1186/s12884-023-05731-1

**Published:** 2023-06-01

**Authors:** Camilla Forbes, Hayley Alderson, Jill Domoney, Alexandra Papamichail, Vashti Berry, Ruth McGovern, Nick Sevdalis, Judith Rankin, Mary Newburn, Andy Healey, Abigail Easter, Margaret Heslin, Gene Feder, Kristian Hudson, Claire A. Wilson, G. J. Melendez-Torres, Louise M. Howard, Kylee Trevillion

**Affiliations:** 1 University of Exeter, Exeter, UK; 2 Newcastle University, Newcastle Upon Tyne, UK; 3 King’s College London, London, UK; 4 Bristol University, Bristol, UK; 5 Improvement Academy, Bradford, UK

**Keywords:** Implementation, hIDVA, Domestic violence, Health, Maternity services

## Abstract

**Background:**

Healthcare-based Independent Domestic Violence Advisors (hIDVA) are evidence-based programmes that provide emotional and practical support to service users experiencing domestic abuse. hIDVA programmes are found to improve health outcomes for service users and are increasingly delivered across a range of healthcare settings. However, it is unclear how hIDVA programmes are implemented across maternity services and the key facilitators and barriers to their implementation. The aim of this study was to identify; how many English National Health Service (NHS) Trusts with maternity services have a hIDVA programme; which departments within the Trust they operate in; what format, content, and variation in hIDVA programmes exist; and key facilitators and barriers of implementation in maternity services.

**Methods:**

A national survey of safeguarding midwives (Midwives whose role specifically tasks them to protect pregnant women from harm including physical, emotional, sexual and financial harm and neglect) within all maternity services across England; descriptive statistics were used to summarise responses. A World Café event (a participatory method, which aims to create a café atmosphere to facilitate informal conversation) with 38 national key stakeholders to examine barriers and facilitators to hIDVA programme implementation.

**Results:**

86/124 Trusts (69%) with a maternity service responded to the survey; 59(69%) of respondents reported that they had a hIDVA programme, and 47(55%) of the hIDVA programmes operated within maternity services. Key facilitators to implementation of hIDVA programmes included training of NHS staff about the hIDVA role and regular communication between Trust staff and hIDVA staff; hIDVA staff working directly from the Trust; co-creation of hIDVA programmes with experts by experience; governance and middle- and senior-management support. Key barriers included hIDVA staff having a lack of access to a private space for their work, insecure funding for hIDVA programmes and issues with recruitment and retention of hIDVA staff.

**Conclusions:**

Despite hIDVA programmes role in improving the health outcomes of service users experiencing domestic abuse, increased funding and staff training is needed to successfully implement hIDVA staff in maternity services. Integrated Care Board commissioning of acute and mental health trust services would benefit from ensuring hIDVA programmes and clinician DVA training are prioritised.

**Supplementary Information:**

The online version contains supplementary material available at 10.1186/s12884-023-05731-1.

## Background

We recognise that people who experience domestic violence and abuse (DVA) use different phrases to describe their experiences e.g., victim, survivor, person with lived experience of domestic abuse. As identified by the Survivor’s Voices Charter [1], many people may not identify with commonly used terms in the academic literature, such as “victim” or “survivor”. They may either not have heard of these terms or do not feel their experiences are adequately summed up by these phrases. For this reason, we use the wording “people who have experienced DVA” throughout this paper. In our service user commentary at the end of this paper, the authors use language with which they identify as individuals.

The numbers of people reporting experiences of DVA globally have been increasing over recent years; in the year ending March 2020, 5% of adults in England (aged 16 to 74 years) reported experiencing past-year DVA (73% female), an increase of 6% from the previous year [2]. Almost two-thirds (73%) of DVA is directed towards women, and it is estimated around 30% of DVA begins during pregnancy [3]; pregnancy is often a turning point for women’s experiences of DVA [4]. Risk factors associated with DVA in pregnancy include unplanned pregnancy and having parents with less than a high-school education [5]. DVA in the perinatal period is associated with several pregnancy and labour complications as well as negative impacts on child developmental outcomes [6, 7]. Therefore, identifying those who experience DVA during pregnancy and connecting them to specialist support is essential to improve outcomes for women and their families. Despite this, there exist multiple barriers for women in disclosing their experiences of DVA [8], and many women do not receive the support they need. Research suggests that women are more likely to disclose DVA in a healthcare setting, particularly in the presence of a trusted professional [3, 9]. In the perinatal period, women are in repeated contact with healthcare services and UK guidance in maternity services includes routine enquiry about DVA by midwives [10]. Therefore, the antenatal period offers a good opportunity to intervene. 

Independent Domestic Violence Advisor programmes aim to secure the safety of those at risk of harm from intimate partners, ex-partners, or family members. They work with those affected by DVA to assess risk, develop safety plans and reduce abuse via emotional and practical help (e.g., providing support in court proceedings, exploring housing options). In healthcare settings, healthcare-based Independent Domestic Violence Advisor (hIDVA) programmes also often include the delivery of DVA training to healthcare staff, alongside the usual advocacy support to people experiencing DVA. In the UK, hIDVA staff receive specialist accredited training and hold a nationally recognised qualification. There is evidence for the effectiveness of hIDVA programmes across a range of healthcare settings [11–13], but less is known about their implementation or impact within maternity services, where many cases of DVA among women are detected. hIDVA programmes have been shown to improve the detection of various levels of DVA risk among service users, and to reduce women’s experience of abuse and further health service use [12]. hIDVA programmes have also been shown to improve staff knowledge and attitudes towards DVA and local DVA detection and care quality [11–13].

A recent three-year evaluation of hIDVA programmes across primary care, acute and mental healthcare settings [13] identified key implementation issues that could influence the outcomes of hIDVA programmes. Issues identified include a demonstrable commitment of healthcare organisations to address issues of DVA, including senior-level buy-in of hIDVA programmes; an understanding and exploration of staff- and organisational-level preconceptions about DVA; ensuring hIDVA staff have opportunities to work directly from healthcare services; formal staff training, combined with informal coaching, to increase staff competencies to address DVA, and effective referral pathways to local DVA agencies [13, 14]. An additional output from this evaluation study was the development of Toolkit guidance for healthcare service providers on how to deliver hIDVA programmes (named the Health Pathfinder Toolkit). This guidance provides recommendations on a range of Trust-level strategies that can be delivered to support the uptake of hIDVA programmes, but there is a lack of detail on specific implementation strategies that can support the successful embedding of hIDVA programmes in maternity services.

Despite evidence for the effectiveness of hIDVA programmes in improving healthcare responses to DVA, there are no centralised funding programmes in England. As a result, the number, scale and content of hIDVA programmes varies across the country. When this study was planned, it was unknown how many English NHS Trusts with a maternity service had a hIDVA programme or how these programmes could be successfully integrated within maternity services. More broadly, it was unclear what key barriers and facilitators exist to implementing hIDVA services in maternity settings.

Therefore, this study aimed to:

1. Map existing hIDVA services, configurations, and implementation factors through a nationwide survey to chart service provision across maternity services in England.
2. Build upon the findings from the survey to generate further insights regarding the barriers and facilitators of implementing hIDVA programmes across maternity service in England, through a World Café (see Methods) stakeholder event.

These two data collection phases are part of a wider project which aims to evaluate current implementation activities of hIDVA programmes within maternity services in England.

## Methods

### Phase 1- Survey

#### Study design

A national cross-sectional survey of Trusts with maternity services in England.

#### Sample/Participants

The target population for this online survey was safeguarding midwives and senior healthcare staff within maternity services across 124 Trusts with maternity services.

#### Recruitment

Participants were purposively sampled by their professional role. Email addresses were sourced by searching Trust websites and through known contacts. Where it was not possible to identify the safeguarding midwives’ contact details, emails were sent to the Trust Head of Midwifery or Head of Safeguarding staff. A maximum of four emails were sent to potential participants sharing the survey link and encouraging completion. To maximise response rates, respondents had the option of completing the survey questions over the phone with researchers, who would input the responses directly onto the online survey.

#### Survey design

The online survey was hosted on the JiSC platform and was open from 27^th^ October 2021 to 28^th^ March 2022. It included 35 brief multiple-choice questions with free-text responses (Additional file 1) and took approximately 5–10 min to complete. Prior to roll-out, the survey was piloted with a small stakeholder group, which included senior research midwives and clinical academic midwives. Feedback included small changes to enhance comprehension of terms used and ensure clarity about the scope of the survey. Recommendations were also provided about the best ways to reach the target population.

The initial page of the survey included information about the wider study as well as the aims of the survey and required respondents to provide their consent to take part. Only those who gave consent were given access to the survey questions.

The survey included the following main sections of questions:

- *Respondent characteristics*: Basic demographic information was collected, including respondents’ email, role, and Trust.
- *DVA policy*: To understand the wider context in which hIDVA programmes operate, respondents were asked about the presence of any Trust DVA strategy/policy, the establishment of staff roles that sought to champion issues of DVA across the Trust and details on DVA training programmes for staff in maternity services. Respondents were also asked if they had heard of the Health Pathfinder Toolkit.
- *IDVA provision*: Respondents were asked whether their Trust had a hIDVA and, if yes, how many and in which services they operated, as well as how they were employed and if they worked directly from the Trust and their (i.e., co-located between the two services) DVA service. Two questions with free-text boxes for responses were asked about factors that supported or acted as barriers to the provision of a hIDVA.

#### Analysis

Descriptive statistics (i.e., mean values) were used to summarise findings and calculations conducted in the JISC platform. Free-text responses to the questions asking about facilitators and barriers to hIDVA programmes were inputted into NVivo, however due to the briefness of the responses, analyses was restricted, therefore responses were categorised by CF.

#### Data cleaning

Only one response per Trust was needed. On the occasion where more than one response was provided within a Trust, the responses were extracted and compared, and the most completed survey responses retained.

### Phase 2- World Café event

#### Study design

A World Café event took place in April 2022 with key stakeholders; due to the ongoing COVID 19 pandemic, the event took place online. The event lasted for 3.5 h and incorporated an overview of the research programme, break-out room discussions of 6 pre-defined open-ended questions, developed by the research team, and a presentation from an established hIDVA programme. The World Café is a participatory method, which aims to create a café atmosphere, which in turn results in the facilitation of informal conversations [15]. The ethos of the World Café style is that it enables a cross-pollination of ideas across heterogeneous groups of individuals and it can facilitate an interactive learning format within which all participants can learn from each other [16]. Participants took part in consecutive rounds of conversations, each focused on pre-set questions of interest to the research team. All participants discussed all questions in order to systematically address topics [17]. At the end of each discussion, a ‘table host’ (i.e., a member of the research study team) summarised the key findings to ensure they captured the important elements of the conversation. This summary was then used to introduce the topic to the next group of individuals, resulting in the previous discussions being constantly added to and mutual reflections taking place [18].

#### Sample/Participants

We purposefully recruited participants to the World Café event who were key stakeholders with experience of delivering hIDVA programmes or individuals who have been in receipt of hIDVA programmes within maternity services in England. For example, we targeted participants who were national maternity service and safeguarding leads, NHS England Maternity Research Leads, commissioners, senior maternity clinical/service leads, Trust safeguarding leads, midwives, hIDVA staff, DVA Coordinators, specialist DVA voluntary sector services, people who have experienced DVA, the Pathfinder toolkit developers.

#### Recruitment

An invitation to participate was advertised on the online event organising platform, Eventbrite. Details of the event were circulated to individuals known to the study team who met the stakeholder profile, individuals who had previously has some involvement with the research project, including respondents of the survey mapping study who agreed to further contact about the study, and established public and patient involvement forums/individuals with lived experience. The event was also advertised on social media platforms (e.g., twitter) and via relevant online DVA networks (e.g., Violence Abuse and Mental Health Network). Individuals were asked to register for the event and were also asked to forward details of the event to their colleagues and peers.

#### World Café event design

In line with the World Café event methodology, participants were split into small groups of between 6–8 people and participated in discussions lasting 20–25 min per question. There were six questions in total (Additional file 2) that explored what core activities were perceived as enabling factors when introducing and implementing hIDVA programmes in maternity services and what potential challenges may hinder hIDVA programmes working well. In each group a table host encouraged participants to contribute to the discussion, listened to contributors, kept the conversation on track and took notes. The table host moved between rooms to ask each group one of the set questions. Each group answered all six questions; at the beginning of each discussion the table host verbally summarised the discussion of the previous group of participants, to facilitate the process of building upon previous discussion points.

#### Analysis

Two researchers (AP and HA) were involved in the analysis of the notes from the World Café event. A codebook thematic analysis was conducted, and followed the six steps identified by Braun and Clarke [19] of: (1) familiarisation of the data; analysis began by researchers (HA and AP) reading the content of each set of notes multiple times to ensure familiarity and immersion in the data. This was followed by (2) individually generating initial codes and highlighting interesting findings within the data. Steps 3–5 included searching for themes and gathering data relevant to each theme; reviewing the themes and finally defining and naming the themes, NVivo12 was used to aid this process. The final step [6] was to relate the themes back to our initial research question and collate them to produce this manuscript. Codebook thematic analysis enabled us to develop a coding framework at the beginning of the analysis process. This framework provided the overall structure for coding, however the researchers refined and developed themes during data analysis as necessary [19].

#### Data checking

As described above, each question was allocated 20–25 min for discussion. At the end of each question the table host summarised the discussion, highlighting the top key points within each group and participants had an opportunity to clarify and/or add information if needed. A summary of findings was also circulated to all World Café participants, following the event, to provide a further opportunity for sense checking the main findings extracted and enabling participants to send feedback/comments and additional information to the team.

## Results

We present the results in two sections. The first section presents the quantitative data findings from the national survey of Trusts and the second section presents the qualitative findings from the free-text responses on the national survey and the notes from the World Café event.

*National survey—*We received a total of 86 responses from the 124 (69%) Trusts contacted. The majority of respondents,* n* = 70(81%), were named safeguarding midwives, seven (8%) respondents were Trust Heads of Safeguarding, seven (8%) were Heads of Midwifery, one was a research midwife, and one was a safeguarding advisor.

*World café event data details—*39 individuals registered for the event and 38 participants attended, including NHS professionals (*n* = 14), specialist DVA voluntary sector professionals (*n* = 8), Commissioners (*n* = 4), individuals with lived experience (*n* = 4) and eight individuals from backgrounds including academics, public participation lead, peer supporters and carers. Of the 14 NHS Professionals, 11 had maternity experience/knowledge and expertise and were in roles such as specialist DVA midwife, Head of Midwifery and/or safeguarding midwife, the remaining three were hospital IDVAs and had a more generic safeguarding role extending outside of the maternity department. Following the World Café event, a summary document of the findings was developed and circulated to all attendees. Feedback was received from five participants, suggesting a few minor alterations but overall reporting that they felt the summary was an accurate reflection of the discussion that occurred at the event.

### Quantitative data findings

**Fig. 1:**
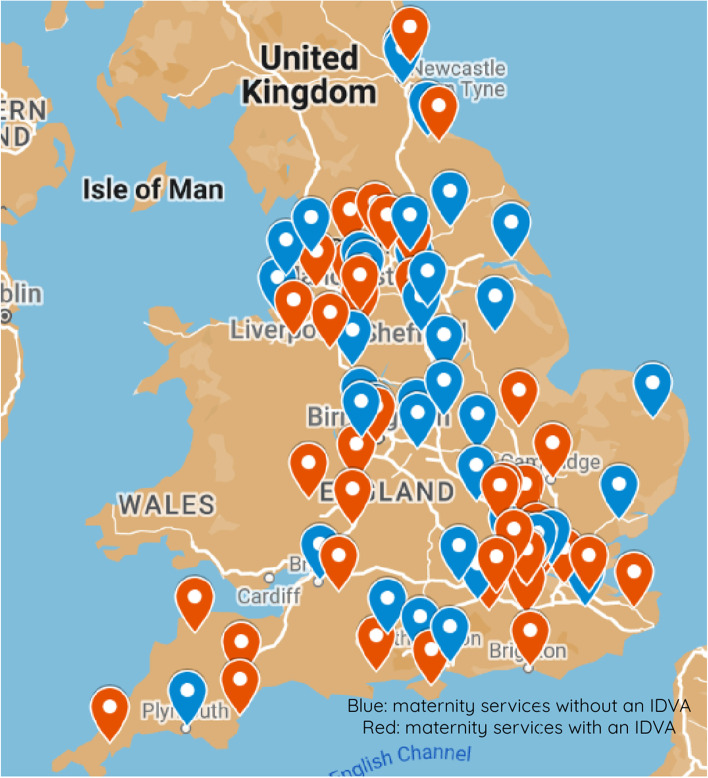
Illustration of the interactive Google My Map of maternity services respondents with or without an IDVA programme. Map data © 2019 GeoBasis-DE/BKG (© 2009), Google

Across all respondents to the national survey, 47(55%) indicated that their Trusts had a hIDVA programme in their maternity service. Figure 1 demonstrates the spread of Trust respondents and those with or without a hIDVA in post, with less hIDVA programmes reported in the East of England and Yorkshire.

### Data cleaning

Six trusts provided multiple responses to the survey; the research team reviewed the responses and the response that was the most complete, with comprehensive free-text, was included. Responses echoed each other and there were no conflicts in the information provided.

### Wider DVA policy

Table 1 summarises responses to survey questions about maternity service or Trust-wide policies regarding DVA. Most respondents said that where their Trust had a domestic abuse policy 70 (90%) it applied to both service users and staff. Five (6%) reported the policy applied only to service users and three (4%) were unsure who the policy covered.

**Table 1:** DVA policy of maternity service and Trust

Has your maternity service/Trust…		Yes	No	Don’t know	other
1	Have a domestic abuse strategy or one in development?	63(74%)	18(21%)	4(5%)	n/a
2	Have a Trust wide, stand-alone domestic abuse policy?	80(93%)	4(5%)	2(2%)	n/a
3	Set-up any domestic abuse champion^a^ roles?	34(40%)	51(59%)	1(1%)	n/a
4	Provided domestic abuse training for maternity service staff?	79(92%)	3(3%)	0	4(5%)

^a^A domestic abuse champion takes a leading role on domestic abuse issues within their setting and acts as a key contact person on issues of domestic abuse both within and outside of that setting. They will be able to advise their colleagues on issues around domestic abuse and ensure that their colleagues are aware of, and have access to, local domestic abuse support resources

DVA training for maternity service staff was mandatory for the majority, and was provided for level three staff^1^[20] on an annual basis and as part of service/safeguarding training. Seven Trusts mentioned that their hIDVA provided this training or additional bespoke training.

A total of 58(67%) Trusts confirmed that they were able to audit rates of routine DVA enquiry by midwives/staff and 65(76%) could provide numbers of clinical referrals to DVA specific risk assessment conferences.

**Fig. 2:**
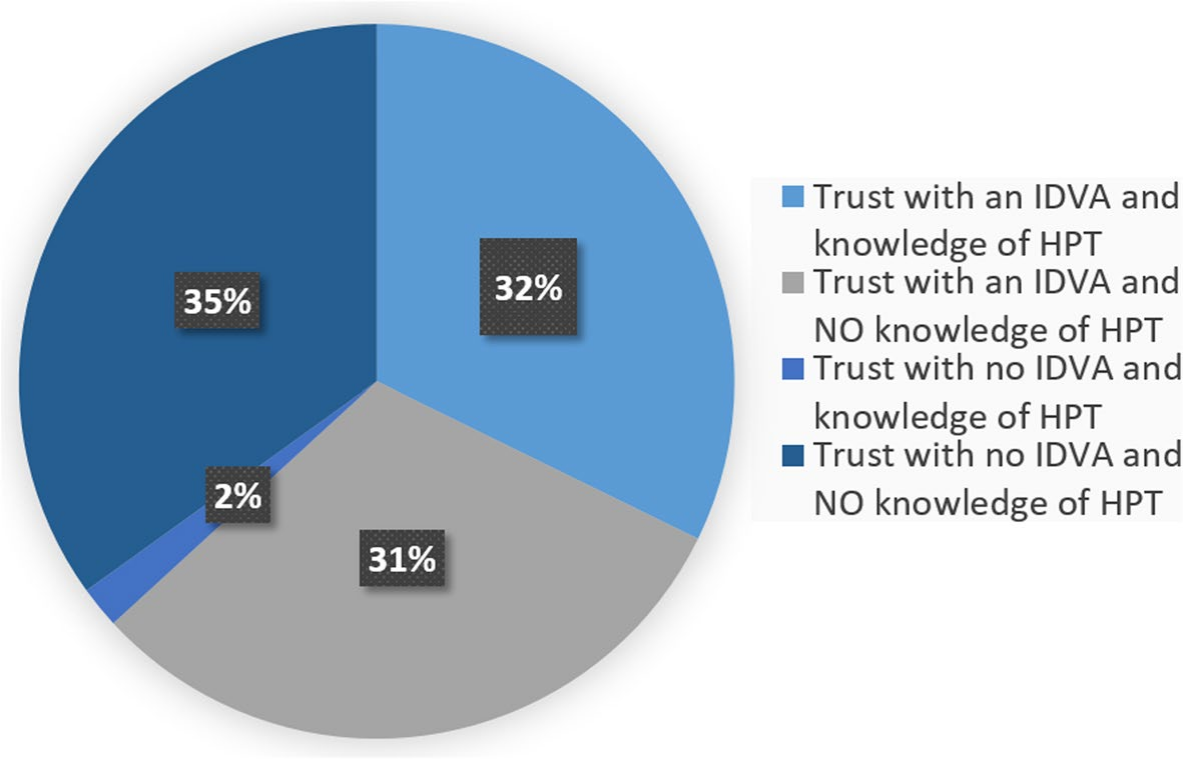
Respondent’s knowledge of Health Pathfinder Toolkit (HPT)

In relation to the Heath Pathfinder Toolkit, 51(58%) respondents reported not having knowledge of the Health Pathfinders Toolkit for DVA. This was evenly spread across Trusts with or without a hIDVA (Fig. 2).

### hIDVA programme provision

Within the 47 Trusts with hIDVA programmes, 28(60%) of the hIDVA staff worked across the whole Trust, with maternity and emergency services identified as the main departments needing the service. Nine Trusts identified that their hIDVA staff also covered community healthcare settings.

**Fig. 3:**
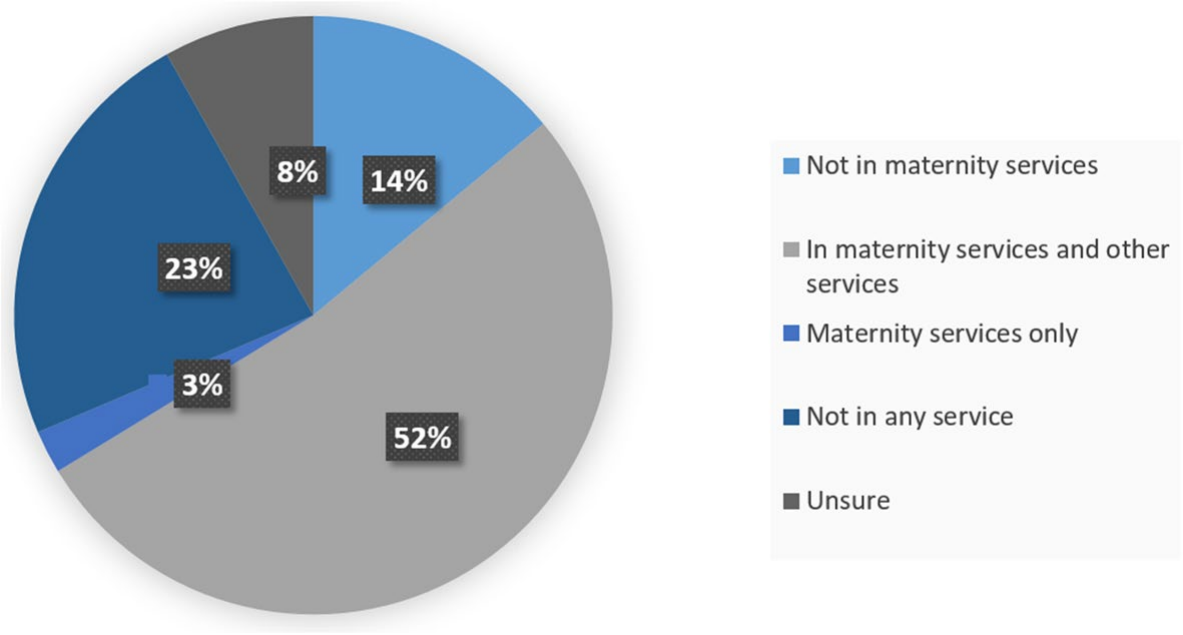
Trust services where IDVAs operate

Fourty five trusts responded to the question of how many hIDVA staff they have in operation, 30(67%) of these had one hIDVA staff member, 13(29%) had two hIDVAs and two Trusts had three (4%) hIDVAs in post (Fig. 3). When asked about how their hIDVA staff were employed, among the 41 respondents to this question, 23(56%) stated that their hIDVA staff were employed directly by a DVA organisation and eight (19%) said they were directly employed by the Trust. Four (10%) stated that the hIDVA staff were seconded to the Trust and 15% identified other means of employment which included other non-healthcare statutory funding sources.

There was variability in how hIDVA staff were funded: nine (20%) of hIDVA staff were funded by the Trust, while 10(22%) were funded by the local authority and six (13%) by the clinical commissioning group.^2^ 11(24%) had other means of funding. The Trusts that had other funding arranged listed, Public health, Police, Ministry of Justice, Mayor’s office, and joint funding arrangements while 10(22%) respondents did not know how their hIDVA staff was funded.

Forty-five Trusts indicated that over half of hIDVA staff (*n* = 25 56%) were co-located between the Trust maternity services and the local domestic abuse service (i.e. they worked directly from the two services as part of their working arrangements), while 6(13%) operated exclusively from their DVA service and nine 9(20%) identified that their hIDVA staff were located solely at the Trust.

The time hIDVA staff had been in post at a Trust varied, with one Trust having had a hIDVA in post for 14 years and seven only having a hIDVA in post a few months. Several respondents were unsure how long the hIDVA staff had been in place. When known, respondents reported that 19(40%) of hIDVA staff were employed permanently and 16(33%) on a temporary basis for no longer than two years.

### Qualitative data findings

This section primarily reflects data collected within the World Café event; however, it also incorporates the data from the free text response to the survey. We have identified when there are discrepancies or agreements between the different data sources. We found two over-arching themes of enablers and challenges when introducing and implementing hIDVA programmes in health care settings. Eight subthemes were developed through the analysis; each one is described in further detail below.

### Enabling factors to support successful implementation of hIDVA services

Factors that facilitated the implementation of hIDVA services included:

#### Awareness raising and training

A recurrent theme mentioned by participants in world café discussions was that there needed to be awareness raising regarding the hIDVA role, to both maternity and other healthcare staff and to individuals who would benefit from the hIDVA service (i.e., people who have experienced DVA). There was agreement that all Trust staff should have the necessary knowledge to accurately introduce the hIDVA programme to service users and to manage expectations about the hIDVA role. For instance, maternity staff should have an understanding that service users’ interaction with a hIDVA staff member is voluntary in nature (i.e., consent must be obtained from service users prior to a referral being made to an hIDVA and services users must be made aware that there is no obligation to use the service); healthcare staff should also be aware of the hours that hIDVA staff are available to make contact with service users.

Participants described that midwives are not confident to obtain consent to refer service users to hIDVA staff if they do not understand the role of the hIDVA programme and they worry about making inappropriate referrals if they do not fully understand their role and how it will benefit the service user. In addition, due to the rotation of staff within healthcare settings, participants explained that there is a need to clearly and continually communicate the remit of the hIDVA role to the changing workforce, and to articulate how the role dovetails alongside existing services. To achieve this, suggestions included that training to staff should be rolled out across the Trust on issues of DVA and the role of hIDVA staff (both within maternity and across the wider Trust where possible). One of the responses to the free text questions in the survey also reported that where high levels of DVA are identified locally, it supported the case for the Trust to have a hIDVA programme, highlighting a cyclical process of raising awareness of DVA more widely among staff, and thereby increasing rates of DVA identification among service users, and the perceived need for a hIDVA in healthcare settings.

#### Co-location of hIDVA staff and visibility in Trusts

Locating hIDVA staff within the Trust and increasing their physical visibility in maternity services were perceived to make the hIDVA role more impactful. When hIDVA staff can attend regular staff meetings with midwives, provide updates regarding referrals they have received and undertake ongoing promotion of their function to staff, this was viewed as pivotal to successful recognition of their role. In addition, hIDVA staff being present on maternity wards and being located in the same physical space as maternity service staff was perceived by participants as enabling greater staff awareness about DVA issues generally as well as the hIDVA role. It was perceived that co-location could also foster joint-working practices between health staff and hIDVA staff more easily than if hIDVA staff were not based at the Trust. Discussions also identified that the physical visibility of hIDVA staff in the Trust to service users was important, as it contributed to reducing stigma around DVA and facilitating open conversations among healthcare staff and service users who have experienced DVA about their experiences of abuse and how they could benefit from the hIDVA programme.

#### Co-creation of hIDVA services with experts by experience

Participants felt strongly that people who have experienced DVA need to be involved in developing and co-designing hIDVA programmes, from the outset of the design stages. Individuals who have experienced DVA and professionals with expertise in DVA can draw on their understanding of what acts as barriers and facilitators to accessing/delivering support which in turn could translate into a service being more responsive to service users’ needs. Discussions in the world café event focused on when the co-creation of hIDVA programmes with people who have experienced DVA was perceived to be necessary. Examples included when communities had specific needs, such ethnically minoritized groups, women with immigrant status, communities requiring support in alternative languages and individuals who need support regarding health literacy. There was also consensus that people who have experienced DVA should be involved on an ongoing ‘long term’ basis to both support the implementation of the hIDVA programme and to respond to the real-world adaptation of the hIDVA programme if needed. A further point of convergence was that people who have experienced DVA that are involved in design hIDVA programmes need to be appropriately remunerated for their time.

### Communication and relationship building

A recurrent theme was that ongoing communication between hIDVA staff and professionals (i.e., both internal Trust staff and community partners) was key both at a practical day-to-day level across healthcare departments, among frontline professionals within the Trust and throughout Trust management hierarchies; this can ensure the hIDVA programme is understood and implemented successfully. Closely linked to this was the identification that hIDVA staff members’ ability to develop relationships both with professional colleagues and people who have experienced DVA was critical for success. Effective communication and relationship building was perceived to be enhanced if hIDVA staff were perceived to be ‘approachable’, ‘understanding’ and working using a trauma-informed approach. Participants suggested that hIDVA staff need to build rapport quickly with people who have experienced DVA, have the skills to adapt quickly to situations that arise in supporting people who have experienced DVA, and to have an awareness that risks of harm from DVA may be much higher for women than originally thought.

### Governance and management support

Participants identified a need for clear Trust governance structures to ensure the hIDVA programme is prioritised within services and among staff. This includes having active support from both middle- and senior-level Trust management, so that hIDVA staff are seen as fulfilling an important function and to ensure funding and support is continued. Participants also described the necessity of having supportive management structures and clear accountability processes in place to oversee the implementation activities of the hIDVA programme.

### Funding and commissioning

Funding and commissioning cycles were recognised as major influences to delivering an hIDVA programme. The survey free-text responses highlight that a range of outside agencies provided piecemeal funding for hIDVA programmes, including Public Health, Ministry of Justice, Police and Crime Commissioner, Local Authority, Clinical Commissioning Groups (CCG) and Mayor’s office. This finding highlights the range of possible organisations involved in the commissioning of hIDVA programmes.

### Challenges that impact the successful implementation of hIDVA programmes

As expected, despite the enablers described above, key challenges around the implementation of hIDVA programmes arose when there was lack of awareness and training among healthcare staff regarding the hIDVA role. This included limited physical visibility of hIDVA staff within maternity services, limited or no arrangements for hIDVA staff to work both within Trusts and from their local DVA service and poor communication and relationship building between hIDVA and Trust staff. In addition, further challenges were discussed, as described below.

### Access to a private room

Participants reported difficulties at times regarding hIDVA staff having access to confidential and ‘safe spaces’ to initiate conversations and conduct assessments with people who have experienced DVA. Difficulties were encountered with respect to locating a private room/space within the hospital/Trust services and the potential for the abusive partner to be present at healthcare appointments, therefore hampering opportunities for confidential conversations to take place.

### Funding, retention, turnover of staff and capacity

Although there is National Institute for Health and Care Excellence (NICE) guidance in place for NHS Trusts to deliver DVA services [21] there are no current statutory requirements for Trusts to have hIDVA programmes in place; the findings of this work highlights that a lack of national government prioritisation for implementing hIDVA programmes acts as a key barrier to endorsing the role. In addition, participants consistently reported the need for hIDVA staff to have more securely funded posts (i.e., not short term/temporary contracts). Participants unanimously agreed that specific longer-term funding for hIDVA staff is necessary to provide a consistent service; it was perceived that protected funding would in turn contribute to lower levels of hIDVA staff turnover and ensure the hIDVA role is viewed as sustainable. Participants reflected that precarious contracts for hIDVA staff, with limited funding periods, do not facilitate collaborative working methods, as hIDVA staff are forced to consider other more secure employment options after a short time of being in post in the hIDVA role. Participants described that the high turnover of hIDVA staff was related to three different problems: short term contracts, low pay, and burnout. The provision of regular clinical supervision and effective line management support was attributed to reducing burnout amongst hIDVA staff. Closely aligned to the issue of retention of hIDVA staff was the recognition that many Trusts did not employ enough hIDVA staff to meet the needs of service users referred to the service. In addition, other resource constraints that acted as barriers included a shortage of locally trained hIDVA staff to employ at the Trust and limited time to write a business case and review processes for the establishment of a hIDVA programme when potential funding calls arose.

## Discussion

To the best of our knowledge, this study provides the most extensive data to date about the availability of hIDVA programmes in Trusts with maternity services. Despite evidence for the effectiveness of hIDVA programmes, survey responses demonstrate that just over half of English NHS Trusts with maternity services have a hIDVA programme in place. This finding highlights a lack of Trusts’ implementation of hIDVA programmes across the country. The varied availability of hIDVA services was recognised within the World Café event, when participants often reported experiences regarding trust wide enablers and barriers rather than factors specific to maternity services. This is unsurprising given that commissioners, DVA voluntary sector partner and individuals in generic safeguarding or IDVA roles had experience of implementing hIDVA programmes at the wider organisational level within healthcare settings. Many of the findings are, therefore, applicable across Trust wide services rather than specific to maternity services. Key facilitators to implementation of hIDVA programmes include regular training of NHS staff about issues of DVA and the hIDVA role, alongside regular communication and joint-working practices between Trust and hIDVA staff; the ability of hIDVA staff to work directly from healthcare Trusts; the co-creation of hIDVA programmes with experts by experience, and adequate governance of hIDVA programmes by Trust middle- and senior-management. Key barriers include hIDVA staff having a lack of access to a private space for their work; insecure funding for hIDVA programmes and issues with recruitment and retention of hIDVA staff.

A national survey by an established DVA third-sector organisation (i.e. SafeLives) of 153 domestic abuse services conducted in 2020/2021 reported that in England and Wales there are only 66% of the required number of hIDVA staff to meet the needs of people who have experienced DVA and only 1 in 10 services had an hIDVA based in a healthcare setting [22]. This level of provision falls below good-practice guidance in the national Health Pathfinder Toolkit, which advises that Trusts have at least two hIDVA staff in place to enable adequate service provision. The findings from this study provide further evidence for the lack of national provision for hIDVA programmes.

Funding for hIDVA programmes in Trust settings was a consistent implementation barrier identified within the survey and the World Café event. Our findings highlight an inconsistency across the country in how hIDVA programmes were funded (i.e., only 20% of hIDVA staff were directly funded by the Trust). This finding is likely to reflect the lack of national commissioning prioritisation for such DVA programmes. There were also concerns raised in the study about the (in)adequacy of the salaries offered to hIDVA staff for this complex and senior role (i.e., around £25,500 per year or NHS band 5), as well as a lack of long-term or sustainable funding for the role (only 40% of respondents identifying that their hIDVA staff member was employed permanently). Finally, the results of this study highlight that numerous different organisations are funding hIDVA programmes on a piecemeal scale across English NHS Trusts. The lack of specific and ring-fenced funding for hIDVA programmes creates significant barriers to the successful implementation of hIDVA services, as well issues with recruiting and retaining hIDVA staff due to short-term, insecure contracts, leading to high levels of staff turnover and increasing the potential for hIDVA staff to experience burnout [22, 23]. With the recent establishment of Integrated Care Boards (ICBs) in England and Wales, who have responsibility for commissioning healthcare services/interventions alongside ensuring such services include involvement of non-statutory community partner agencies, there is a great opportunity to set a clear prioritisation for the delivery of healthcare DVA interventions with sustainable co-commissioned arrangements between relevant statutory and non-statutory agencies. The hIDVA programme is a prime example of a partnership between statutory and non-statutory agencies to improve peoples’ health outcomes and, therefore, lends itself to sustainable jointly commissioned funding arrangements. Examples of jointly funded healthcare programmes for people who have experienced sexual violence are already in existence and demonstrate positive healthcare outcomes for service users [24]. The findings reported from this study align with those reported in the 2020/2021 SafeLives survey, where 37% of respondents stated that recruiting and retaining hIDVA staff was difficult due to inconsistent and short-term contracts [22]. A further key finding of this study is a lack of locally trained hIDVA staff for healthcare services to employ, suggesting the need for more funding to train a workforce of hIDVA staff and the potential benefit of national IDVA training organisations to scope out regional gaps in workforce availability and to promote and support the training of staff in local DVA services in these areas.

Healthcare staff training regarding DVA and hIDVA staff roles was also highlighted as integral to the successful implementation of hIDVA programmes. This finding aligns with other research which highlights a lack of DVA training among staff across a range of healthcare settings, including primary care, acute services and mental health services and can result in an under-detection of cases of DVA among service users [8, 25–27]. In this study, DVA training was perceived as necessary to raise awareness of the importance of the hIDVA programme role, in promoting the complimentary nature of the hIDVA staff work alongside existing healthcare services and in ensuring that the hIDVA service is utilised.

Most survey respondents (58%) reported being unaware of the Health Pathfinder Toolkit guidance on implementing hIDVA programmes in NHS settings. Most respondents were safeguarding midwives, a role where you might expect this awareness and knowledge. However, it may be that the Trust safeguarding leads or DVA champions were aware and were not surveyed here. In addition, our survey only asked about awareness of the toolkit; therefore, we are mindful that respondents may be aware of the toolkit but are not implementing it or indeed they may be implementing many elements of good practice highlighted within the Toolkit without specifically having knowledge of these points. Further research would benefit from identifying ways to increase healthcare staff awareness of the Toolkit, to evaluate staff views about the guidance recommendations, and to examine their experience of utilising the Toolkit to implement hIDVA programmes.

Further implementation barriers identified in this study are a lack of national policy priority and guidance for hIDVA programmes and a lack of Trust recognition and support for the hIDVA role. This finding aligns with a 2016 national DVA sector survey, which reported that only 5% of services agreed that the UK government recognised the importance of the role that IDVAs provide [28]. These findings signify a need to raise the profile of hIDVA programmes both at a local and national level. This could be led by governmental policy makers, relevant independent domestic abuse services and via the newly established ICB partnerships. It is also important to recognise that hIDVA programmes are novel in their hybrid model approach, which draws on the expertise of staff from local DVA sector services to deliver DVA interventions within healthcare services. This approach acknowledges the skills and experience of non-statutory community partners in helping to improve health outcomes for service users and fosters shared learning between statutory and non-statutory service providers. It is important, therefore, for the successful implementation of hIDVA programmes that the two partner services foster mutual respect, through both joint-working practices and shared funding agreements.

A recent study by Hegarty et al. [29] identified that a healthcare practitioners ability and readiness to address DVA is only realised when the health system is equipped to manage DVA. This requires support through DVA policies and procedures alongside the required infrastructure and culture to effectively address issues of DVA among service users. However, this study highlights that current contexts in NHS Trusts are not always conducive to effective hIDVA programme implementation. More is needed around local policy development around DVA, alongside increased training of healthcare staff, including DVA awareness raising. hIDVA programmes are likely to fail if the context is not receptive to identifying and supporting DVA at a broader organisational level.

### Strengths and limitations

Despite having a reasonable response rate, particularly in light of the fact that data collection ran during the COVID-19 pandemic and over a winter period which sees the NHS under increased demand, 30% of eligible Trusts did not participate in the survey. However, there was a good spread of responses across different geographical regions in England (Fig. 1); there was also 100% completion rate of the survey items by those who participated in the survey.

It is important to note that whilst participants had specialist experience of working in maternity settings and were specifically asked questions related to hIDVA provision within maternity departments, many responses to questions at the world café event were broad in scope and often described enablers and barriers to implementation of IDVA programmes at the wider organisational level within healthcare settings. Many of the findings are, therefore, applicable across Trust wide services rather than specific to maternity services. However, this paper reports a sub-study from a bigger research programme [30] which will include detailed case study evaluations of hIDVA programmes in 3 NHS Trusts and their experiences, including successes and challenges, of successfully embedding the programme within maternity services. This case study evaluation will allow us to generate more specific guidance around implementation activities for the benefit of maternity service practice.

A limitation of the World Café methodology that we applied was that the findings were derived from written summaries collected by the research team, rather than via verbatim responses from participants (taken from audio-recorded discussions of the World Café conversations). In addition, only five respondents provided feedback on the event summary. However, a key strength of the World Café approach is that it provided an opportunity to bring together a diverse group of people, encapsulating a range of views from different perspectives (e.g., people who have experienced DVA, hIDVA staff, healthcare and midwifery staff and commissioners), to discuss key areas of importance to them in a more flexible, less formalised format than a focus group interview set-up. This research method also enabled ‘cross fertilisation’ of ideas as all participants explored the same topics, and this resulted in a depth of data collected.

## Conclusion

Given the evidence for hIDVA programmes in improving the health outcomes of service users experiencing DVA and staff responses to DVA, there is a need to establish sustainable funding sources for this intervention and to ensure ongoing training of healthcare staff around issues of DVA. This could be realised through long-term jointly funded local DVA community and statutory NHS services delivering hIDVA programmes, as well as the championing of DVA training for healthcare staff within the newly established Integrated Care Boards.

### Service user commentary (Mary Newburn and DS)

This paper demonstrates the patchy provision of professional domestic abuse services for pregnant women whose lives are blighted by a constant threat of violence, fear, anxiety and social stigma. As survivors of domestic violence, only one of us had access to IDVA services. DS found her IDVA well joined up with the maternity services. DS’s partner did not attend her prenatal appointments and so she could talk about what she was going through at home, and she got help leading to her getting out of the situation. Had her partner been at appointments there would have been no way to mention what was going on. Providing private, women-only spaces in clinics is essential. We would have liked to see more detail included about ways NHS trusts and IDVAs communicate about domestic abuse support, such as pull-off stickers on posters in the female toilets that a woman can discreetly put on her notes to signal her need to the staff, unobserved. We would welcome sharing of best practice in providing safe and accessible support for vulnerable women. Crucially, we want to hear the voices of Black, Asian and White women who have used the services, and to hear what works for them.

We are concerned that in many trusts there is no permanent arrangement to fund IDVA posts. Lack of funding and short-term contracts really undermine provision. IDVAs can form a bridge between community-based specialist domestic abuse services and acute-sector maternity services. Joined up health and social care is important at all stages of life, but it cannot happen without staff to do the communication and liaison, to provide training and signposting to help with housing, and legal and benefits advice. The paper makes explicit the staff time and expertise required, and how short-term funding leads to disjointed services and lost expertise. Long-term funding is vital to ensure high-quality services are provided consistently across all trusts without interruption.

## Supplementary Information

**Additional file 1. **

**Additional file 2. **

## Data Availability

The datasets generated and/or analysed during the current study are not publicly available due to the sensitive and personal nature of the material but are available from the corresponding author on reasonable request.

## Footnotes

1 Level three training is aimed at clinical staff who could potentially contribute to assessing, planning, intervening, and evaluating the needs of a person where there are safeguarding concerns.

2 Clinical Commissioning groups (CCGs) are groups of general practices (GPs) which come together in each area to commission the best services for their patients and population.
