# Supplementary material for: A survey and stakeholder consultation of Independent Domestic Violence Advisor (IDVA) programmes in English maternity services
Source: BMC Pregnancy Childbirth. 2023 Jun 1;23:404. doi: 10.1186/s12884-023-05731-1 (PMC10233538; doi:10.1186/s12884-023-05731-1)
Supplement: Supplementary file 1 — Additional file 1. [file 12884_2023_5731_MOESM1_ESM.pdf]

Evaluating models of health-based Independent Domestic Violence Advisor (IDVA) provision within maternity settings

Page 1

What is this request about?

We are asking you to take part in a brief survey, which should take around 5-10 minutes to complete.

What is this study about?

This national research programme is funded by the National Institute for Health Research (NIHR) Applied Research Collaboration (ARC) and seeks to evaluate the implementation of health-based Independent Domestic Violence Advisor (IDVAs) models within English NHS settings that include maternity services.

An IDVA is a trained professional who works to address the safety of people at high risk of harm from intimate partners, ex-partners or family members. They provide emotional and practical support (e.g. general advocacy, safety planning, support with accomodation, finances and court processes). In general, they support people for an average of 2-3 months.

The research programme has three parts:

(1) A national mapping of existing health-based IDVA models within English NHS Trusts.

(2) A stakeholder event which aims to generate guidance on how to facilitate the successful implementation of health-based IDVA models in NHS settings with maternity services

(3) A case study of three NHS Trusts that implement an IDVA model, to evaluate the implementation of the model and the impact of the model in achieving positive service change on staff practice and service user outcomes.

This survey is the first part of our study, a national mapping of existing health-based IDVA models. We are inviting NHS Trusts across England to provide us with information about their domestic abuse strategies/policies and their IDVA provision, if available.

If you choose to take part in this online survey you will be asked to answer some questions on your Trust's provision of a IDVA and/or their domestic abuse strategy/policy. As part of participation, you will be asked to provide your Trust name and your job title. We also ask you to provide your work email address. We are asking for this so that we can contact you in the future about the study, including potentially asking about your interest in taking part in some other parts of this work. If you do not want to hear from us further, leave this question blank.

Do I have to take part?

Participation is completely voluntary. You should only take part if you want to; choosing not to take part will not disadvantage you in anyway. If you choose to take part you will be asked to provide your consent. To do this you will be asked to indicate that you have read and understood the information provided here and that you consent to your data being used for the purposes explained below.

You are free to withdraw at any point during completion of the survey, without having to give a reason. If you wish to 'erase' your responses before exiting the survey, you may need to backtrack through the survey to do this manually. Withdrawing from the study will not affect you in any way. Once you submit the survey, you can contact us to request your data be withdrawn by 31st December 2021, wherein the data will be anonymised. Please do not include any personal identifiable information in your survey responses, except for providing your job title and email address when requested.

What will happen to the results of the project?

The results of the project will be summarised in academic journals, conference presentations and policy briefs. All published work will be anonymised and it will not be possible to identify you or your individual responses in any outputs.

Data handling and confidentiality

Your data will be processed under the terms of UK data protection law (including the UK General Data Protection Regulation (UK GDPR) and the Data Protection Act 2018).

Your data will be held securely at JiSC online survey repository within the EU in accordance with GDPR requirements (please see https://www.onlinesurveys.ac.uk/ for

further information). When you complete the survey, your name, job title, work and email address will be linked to your survey responses. At the end of the data collection period, your survey responses will be downloaded directly from JiSC and stored on a secure server hosted by King’s College London (via a Microsoft Teams account); access to the account will be restricted so that only the study team can access the files. Following download, all data will be deleted from the JiSC survey repository and your responses to the survey questions will be anonymised by separating out your identifiable personal data from your responses. Your identifiable personal data will then be stored in a separate file on the secure Microsoft Teams account, which will only be accessible to the study team. Your personal information will not be shared with any third party.

All identifiable personal data will be deleted upon completion of the project. We will keep your anonymised survey responses for four years following the completion of the study, in line with King's College London’s Data Retention Schedule

Data Protection Statement

If you would like more information about how your data will be processed under the terms of UK data protection laws please visit the link below:

https://www.kcl.ac.uk/research/support/research-ethics/kings-college-london-statement-on-use-of-personal-data-in-research

If you have any questions or require more information about this project, please contact me using the following contact details: Kylee Trevillion at kylee.trevillion@kcl.ac.uk

This survey has been approved by the King's College Ethics Committe reference number: MRA-20/21-26162.

If you are happy to take part in this survey please click the next button to consent.

Consent

1. I confirm that I have read and understood the information for the above project. I have had the opportunity to consider the information and asked questions which have been answered to my satisfaction. ✱ *Required*

- [ ] Yes
- [ ] No

2. I consent voluntarily to be a participant in this project and understand that I can refuse to take part and can withdraw from the project at any time, without having to give a reason. ✱ *Required*

- [ ] Yes
- [ ] No

3. I consent to the processing of my personal information for the purposes explained to me in the information above. I understand that such information will be handled under the terms of UK data protection law, including the UK General Data Protection Regulation (UK GDPR) and the Data Protection Act 2018. ✱ *Required*

- [ ] Yes
- [ ] No

4. I understand that my information may be subject to review by responsible individuals from the College for monitoring and audit purposes. ✱ *Required*

- [ ] Yes
- [ ] No

5. I understand that confidentiality and anonymity will be maintained, and it will not be possible to identify me in any research outputs. ✱ *Required*

- [ ] Yes
- [ ] No

6. I agree that the research team may use my data for future research and understand that any such use of identifiable data would be reviewed and approved by a research ethics committee. ✱ *Required*

- [ ] Yes
- [ ] No

7. I agree to be re-contacted in the future by the study researchers regarding this project. ✱ *Required*

- [ ] Yes
- [ ] No

By clicking **next** you agree to take part in this online survey

Information about you

8. What is the name of the Trust that you work for? ✱ *Required*

9. What is your job title? ✱ *Required*

10. Please state your email address

IDVA provision

11. Do you have any Independent Domestic Violence Advisors (IDVAs) in operation at your maternity service?

IDVA provision.1

12. Based on your experience, please list up to 5 factors that allow your service to have an IDVA in operation (these could be related to commissioning, Trust-level factors, or factors within your service).

13. Which parts of the service do the IDVA(s) operate in (e.g. maternity wards, birthing suites)?

14. How many IDVAs does your maternity service have?

IDVA provision.2

15. Based on your experience, please list up to 3-5 barriers for not having an IDVA in your maternity service

Routine clinical data

As part of the wider study we are planning to evaluate whether IDVA provision impacts on outcomes such as rates of disclosure of domestic abuse. Please use the questions below to let us know if your service may be able to provide data on these outcomes.

16. Could your maternity service/Trust provide anonymised summaries of data on how many women accessing maternity services in the past year have disclosed domestic violence and abuse (subject to permissions)?

16.a. If you selected Other, please specify:

17. Could your maternity service/Trust provide anonymised summaries of the numbers of premature deliveries, low-birth weight and small for gestational age birth outcomes in the past year (subject to permissions)?

17.a. If you selected Other, please specify:

18. Could your maternity service/Trust provide data on the number of people referred from maternity services to Multi-Agency Risk Assessment Conferences (subject to permissions)?

18.a. If you selected Other, please specify:

19. Would you be happy to talk to the research team about anonymously sharing data?

IDVA provision.03

20. Does your Trust have IDVA(s) in operation in any other services?

IDVA provision.3

21. How are the IDVA(s) employed while working with your service?

21.a. If you selected Other, please specify:

22. What is the whole time equivalent (WTE) of the IDVA(s) in your maternity service?

23. How long have they been in operation?

24. Are the IDVAs co-located between the maternity service and their domestic abuse service or do they operate exclusively from their domestic abuse service?

24.a. If you selected Other, please specify:

25. How are the IDVAs funded?

25.a. If you selected Other, please specify:

26. Do IDVAs receive any support from your maternity service with their continuing professional development?

IDVA provision.4

27. Are IDVAs funded on a temporary or permanent basis?

27.a. If you selected Other, please specify:

Temporary IDVA contract

28. If temporary, how long are they funded for?

Domestic Abuse Maternity Service Responses

29. Does your maternity service have a domestic abuse strategy or one in development?

30. Has your maternity service set-up any domestic abuse champion roles?

31. Does your maternity service provide domestic abuse training for maternity service staff?

31.a. If you selected Other, please specify:

DVA training details

32. Please describe how often the training takes place, which staff groups receive it and who delivers it?

Domestic Abuse Trust Responses.2

33. Does your Trust have a stand-alone domestic abuse policy?

DVA Trust policy

34. Does the policy cover patients, staff or both?

Pathfinders Toolkit

35. Have you heard of the health Pathfinders Toolkit for domestic abuse?

Comments

36. Is there anything else you would like to add about your maternity service's approach to domestic abuse?

By clicking **FINISH** your responses to the survey will be submitted

Final page

Thank you for completeing this survey, your responses will be so valuable in enabling us to map the health-based Independent Domestic Violence Advisor (IDVA) provision within maternity services across England.

If this project has harmed you in any way or if you wish to make a complaint about the conduct of the project you can contact King's College London using the details below for further advice and information: The Chair of the Psychiatry, Nursing & Midwifery Research Ethics Sub-committee: **rec@kcl.ac.uk**.

Key for selection options

11 - Do you have any Independent Domestic Violence Advisors (IDVAs) in operation at your maternity service?

Yes
No
Don't Know

14 - How many IDVAs does your maternity service have?

1
2
3
4
5 or more

16 - Could your maternity service/Trust provide anonymised summaries of data on how many women accessing maternity services in the past year have disclosed domestic violence and abuse (subject to permissions)?

Yes
No
Don't Know
Other

17 - Could your maternity service/Trust provide anonymised summaries of the numbers of premature deliveries, low-birth weight and small for gestational age birth outcomes in the past year (subject to permissions)?

Yes
No
Don't Know
Other

18 - Could your maternity service/Trust provide data on the number of people referred from maternity services to Multi-Agency Risk Assessment Conferences (subject to permissions)?

Yes
No
Don't Know
Other

19 - Would you be happy to talk to the research team about anonymously sharing data?

Yes
No
Don't Know

20 - Does your Trust have IDVA(s) in operation in any other services?

Yes - in other services and NOT in maternity service
Yes - in other services AND in maternity service
No - only in maternity services
No - not in any Trust services
Don't Know

21 - How are the IDVA(s) employed while working with your service?

Employed directly by the Trust
Seconded to the Trust
Employed directly by their domestic abuse organisation
Other

24 - Are the IDVAs co-located between the maternity service and their domestic abuse service or do they operate exclusively from their domestic abuse service?

Co-located
Exclusively domestic abuse service
Don't Know
Other

25 - How are the IDVAs funded?

Funded by the Trust
Funded by the Local Authority
Funded by the CCG
Don't Know
Other

26 - Do IDVAs receive any support from your maternity service with their continuing professional development?

Yes
No
Don't Know

27 - Are IDVAs funded on a temporary or permanent basis?

Temporary
Permanent
Don't Know
Other

29 - Does your maternity service have a domestic abuse strategy or one in development?

Yes
No
Don't Know

30 - Has your maternity service set-up any domestic abuse champion roles?

Yes
No
Don't Know

31 - Does your maternity service provide domestic abuse training for maternity service staff?

Yes
No
Don't Know
Other

33 - Does your Trust have a stand-alone domestic abuse policy?

Yes
No
Don't Know

34 - Does the policy cover patients, staff or both?

Patients
Staff
Both
Don't Know

35 - Have you heard of the health Pathfinders Toolkit for domestic abuse?

Yes
No
