# Supplementary material for: A survey and stakeholder consultation of Independent Domestic Violence Advisor (IDVA) programmes in English maternity services
Source: BMC Pregnancy Childbirth. 2023 Jun 1;23:404. doi: 10.1186/s12884-023-05731-1 (PMC10233538; doi:10.1186/s12884-023-05731-1)
Supplement: Supplementary file 2 — Additional file 2. [file 12884_2023_5731_MOESM2_ESM.docx]

Additional file 2- World Café event Questions

Q1- What do you think are the ‘must-haves’ for an IDVA model to work for service users? And are there particular things you would do or have done to ensure these ‘must-haves’ are available?

Q2- What do you think are the ‘must-haves’ for an IDVA model to work for maternity services and are there particular things you would do or have done to ensure these ‘must-haves’ are available?

Q3- What do you think might get in the way of the IDVA model working well?

Q4- How do you think the core characteristics of the IDVA model might need to be tailored to fit service user and maternity service needs?

Q5- In your view, which Pathfinder recommendations feel particularly important for service users and maternity services? Are any topics not covered or not detailed enough?

Q6- What aspects of the IDVAs’ relationship with other Health Professionals and with service users are critical to the success of the model?
